# The Effects of Early Life Stress, Postnatal Diet Modulation, and Long-Term Western-Style Diet on Later-Life Metabolic and Cognitive Outcomes

**DOI:** 10.3390/nu12020570

**Published:** 2020-02-22

**Authors:** Maralinde R. Abbink, Lidewij Schipper, Eva F.G. Naninck, Cato M.H. de Vos, Romy Meier, Eline M. van der Beek, Paul J. Lucassen, Aniko Korosi

**Affiliations:** 1 Brain Plasticity Group, Center for Neuroscience, Swammerdam Institute for Life Sciences, University of Amsterdam, 1098 XH Amsterdam, The Netherlands; maralinde@live.nl (M.R.A.); romymeier93@gmail.com (R.M.); P.J.Lucassen@uva.nl (P.J.L.); 2 Danone Nutricia Research, 3584 CT Utrecht, The Netherlands; Lidewij.Schipper@danone.com (L.S.); Eline.VANDERBEEK@danone.com (E.M.v.d.B.); 3 Department of Pediatrics, University Medical Centre Groningen, University of Groningen, 9713 GZ Groningen, The Netherlands

**Keywords:** early-stress, postnatal-dietary lipids, cognition, neurogenesis, western style diet, metabolism

## Abstract

Early life stress (ES) increases the risk to develop metabolic and brain disorders in adulthood. Breastfeeding (exclusivity and duration) is associated with improved metabolic and neurocognitive health outcomes, and the physical properties of the dietary lipids may contribute to this. Here, we tested whether early life exposure to dietary lipids mimicking some physical characteristics of breastmilk (i.e., large, phospholipid-coated lipid droplets; Concept Nuturis® infant milk formula (N-IMF)), could protect against ES-induced metabolic and brain abnormalities under standard circumstances, and in response to prolonged Western-style diet (WSD) in adulthood. ES was induced by exposing mice to limited nesting material from postnatal day (P) 2 to P9. From P16 to P42, male offspring were fed a standard IMF (S-IMF) or N-IMF, followed by either standard rodent diet (SD) or WSD until P230. We then assessed body composition development, fat mass, metabolic hormones, hippocampus-dependent cognitive function, and neurogenesis (proliferation and survival). Prolonged WSD resulted in an obesogenic phenotype at P230, which was not modulated by previous ES or N-IMF exposure. Nevertheless, ES and N-IMF modulated the effect of WSD on neurogenesis at P230, without affecting cognitive function, highlighting programming effects of the early life environment on the hippocampal response to later life challenges at a structural level.

## 1. Introduction

The early perinatal period forms a sensitive time window for metabolic programming and brain development. Stress exposure during this time (early life stress; ES) has a long-lasting impact on health outcomes, increasing the vulnerability to both metabolic and mental disorders in adulthood [1,2]. Clinical research has confirmed that ES enhances the occurrence of obesity [1,3] and metabolic syndrome [4], as well as the risk to develop cognitive impairments [5,6] and psychopathology [2,7] in adulthood. Contributing to the adverse health outcomes in the current society is the high prevalence of consumption of unhealthy “Western-style” diet (WSD) across the lifespan, which also increases the risk for the development of these disorders [8,9,10,11,12,13]. In fact, WSD affects metabolic and cognitive functioning [14,15] and in addition, previous ES exposure might aggravate negative health outcomes induced by WSD [16,17,18,19]. For example, ES in rodents enhances predisposition for diet-induced obesity in adulthood [16,17,18,19], and while ES leads to a leaner phenotype under standard diet (SD) conditions, ES mice accumulate more fat when exposed to WSD later in life, suggesting increased metabolic vulnerability [19].

The quality of nutrition early in life is increasingly acknowledged as an important determinant of later-life health [20,21], and negative health outcomes induced by ES or WSD are potentially modified by early life diet. Nutritional interventions during infancy and childhood may therefore be considered as potential therapeutic strategies to protect against the manifestation of later metabolic problems [22] and to prevent the negative effects of ES on brain structure and function [23,24]. In line with this, exclusivity and duration of breastfeeding, as compared with formula feeding, are positively associated with later-life metabolic profile [25,26,27] and cognitive performance [28,29,30]. There are many differences between breastfeeding and formula feeding that may contribute to these effects, including differences in nutritional quality of human milk and infant milk formula (IMF). For instance, the physical properties of dietary lipids may be considered as an important modulator of later-life health [31]. Mammalian milk contains large lipid droplets covered by a phospholipid trilayer (milk fat globule membrane; MFGM), consisting of phospholipids, membrane proteins, and cholesterol [32]. In contrast, lipid droplets in current commercially available IMF are small and lack the complex surface area characteristics [33]. The relevance of physical structure of lipids to later life health can be studied by use of a novel concept that resembles the physical properties of lipid droplets in mammalian milk more closely, i.e., containing large lipid droplets surrounded by phospholipids (Nuturis®) [34]. Early life nutritional intervention with Nuturis® in mice attenuates excessive body fat accumulation induced by WSD in adulthood [35,36] and improves specific cognitive functions in adolescent and adult mice [37]. 

Given the positive effects observed of early life feeding with Nuturis® on metabolic and cognitive outcomes and the fact that both outcomes are affected by ES [19,38], the aim of our study is to investigate whether a nutritional intervention with Nuturis® could potentially modulate ES-induced alterations in later life. We set out to study whether an early life dietary intervention with Nuturis® protects against the effects of ES on metabolic health, hippocampus-dependent cognition, and the generation of new hippocampal neurons (i.e., neurogenesis), parameters of brain function and plasticity that are modulated by both ES [38,39,40,41,42,43] and adult WSD exposure [14,39,44,45,46]. This study will not only help to elucidate whether a combination of ES and later-life WSD aggravates health outcomes, but may also reveal whether an early life nutritional intervention in the form of altered physical properties of dietary lipids can mitigate these effects both centrally and peripherally. We here show that the protective and harmful effects of respectively N-IMF and ES on fat accumulation in response to WSD are transient and disappear after prolonged WSD exposure. Furthermore, we show that both ES and N-IMF modulate the WSD-induced hippocampal neurogenic response.

## 2. Materials and Methods 

### 2.1. Animals and Breeding

All C57Bl6j mice were kept under standard housing conditions (temperature: 20–22 °C, humidity: 40%–60%, standard 12/12 h light/dark schedule, chow and water ad libitum). Female 10-week-old mice and 8-week-old male mice were used for breeding in house. All experiments were approved by the Animal Experiment Committee of the University of Amsterdam and performed in accordance to European Union (EU) directive 2010/63/EU.

### 2.2. ES Paradigm

The ES paradigm consisted of limiting nesting and bedding material from P2–P9 as described previously [38,47]. Briefly, at P2, litters were culled to 6 pups/dam and randomly assigned to control (CTL) or ES condition. From P2 until P9, CTL litters (n = 15) received standard nesting and bedding material and ES litters (n = 15) were placed on a fine-gauge stainless steel mesh positioned 1 cm above the sawdust-covered cage floor with reduced amounts of nesting material. Maternal care was observed from P3 to P8 as previously described [24,38]. Mice were weighed at P2 and P9, and male mice were weaned at P21, housed in groups of 2 to 4 animals per cage, and used for experiments. 

### 2.3. Experimental Diets

**Figure 1:**
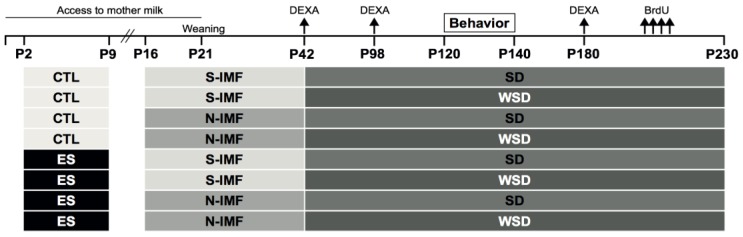
Experimental design. From P2 to P9, mice pups were exposed to early-life stress (ES) or control (CTL) condition. Between P16 and P42, animals were exposed to standard IMF diet (S-IMF) or Concept Nuturis® IMF diet (N-IMF) and from P42 to P230, mice received standard chow diet (SD) or moderate western style diet (WSD). Dual-energy X-ray absorptiometry (DEXA) scans were performed on P42, P98, and P180. Cognitive functioning was tested in behavioral tasks between P120 and P140. Between P190 and P200, mice received two 5-bromo-2’-deoxyuridine (BrdU) injections per day for four consecutive days. Animals were sacrificed 4 weeks after the last injection. The experimental groups are represented below the timeline.

All diets were semisynthetic (Ssniff-Spezialdiäten GmbH, Soest, Germany) and had a macro and micronutrient composition according to AIN-93G-purified diets for laboratory rodents [48]. Specific composition of each diet was described previously [49]. Dams were fed AIN-93G diet throughout breeding, pregnancy, and, partially, the lactation period. From P16 to P42, litters were randomly allocated to standard infant milk formula diet (S-IMF) or Concept Nuturis® IMF diet (N-IMF) initially, resulting in the following four experimental groups: CTL S-IMF n = 25, CTL N-IMF n = 25, ES S-IMF n = 25, ES-N-IMF n = 23. The IMF diets contained 28.3% w/w S-IMF or N-IMF, providing all of the fat in the diet, and were complemented with protein and carbohydrates to match AIN-93G composition. The S-IMF and N-IMF differed in physical characteristic of lipid droplets (i.e., large and phospholipid coating in N-IMF) due to the addition of bovine MFGM-derived phospholipids and altered processing of N-IMF. IMF diets were daily provided as dough on the cage floor in order to preserve lipid structure [37]. Until P21, pups also had access to their own mothers’ milk. Exposure to IMF diet was continued until P42; thereafter, mice were switched to either standard chow diet (SD: AIN-93M) or moderate WSD chow consisting of 22% w/w fat (22% w/w lard, 0.1% w/w cholesterol) until the end of the experiment, resulting in the following eight experimental groups: CTL S-IMF n = 12, CTL N-IMF n = 12, ES S-IMF n = 13, ES-N-IMF n = 11, CTL S-IMF WSD n = 13, CTL N-IMF WSD n = 13, ES S-IMF WSD n = 12, ES-N-IMF WSD n = 12 (see Figure 1). 

### 2.4. Body Composition

Body composition development during WSD challenge was assessed at P42, P98, and P180 using dual-energy X-ray absorptiometry (DEXA scan; Lunar PIXImus) (a small subset of this data was previously reported in [19] fat percentage of CTL and ES S-IMF WSD mice up to P98). Due to technical issues, the P180 scan could only be performed in a subgroup of animals (CTL S-IMF WSD n = 4, CTL N-IMF WSD n = 4, ES S-IMF WSD n = 5, ES-N-IMF WSD n = 4). After rapid induction (<5 min) of anesthesia with a mixture of 5% isoflurane-oxygen (2 l/min), mice were placed on the scanning bed in prone position and supplied with 2% isoflurane-oxygen (2 l/min) for maintenance of anesthesia during the scan. Data are displayed as the relative change (delta) in body weight (BW), lean mass, fat mass, and fat percentage from P42 onwards.

### 2.5. Behavioral Testing

To assess cognitive functioning at P120, a battery of three behavioral tests was conducted: the object recognition task (ORT), object location task (OLT), and T-maze task. Behavioral tests were carried out in the active phase (reversed 12/12 h light/dark schedule), recorded by Ethovision (Noldus), and scored manually using Observer (Noldus). Behavioral tests were performed as described previously [24,38]. 

### 2.6. BrdU Injections

Seven to eight weeks after behavioral testing (P190–P200), animals were injected with 5-bromo-2’-deoxyuridine (BrdU, Sigma-Aldrich), which incorporates in the DNA of dividing cells and allows studying survival of adult-born neurons. BrdU (10 mg/mL dissolved in sterile saline + 0.007M NaOH) was injected intraperitoneally twice a day, for four consecutive days. Animals were sacrificed 4 weeks following the final injection (P230; the experimental timeline displayed in Figure 1). 

### 2.7. Tissue Collection

Mice were fasted for 4 hours prior to sacrifice and subsequently anesthetized via intraperitoneal injection of pentobarbital (120 mg/kg Euthasol®). Upon sacrificing, vena cava blood was collected in EDTA-coated tubes (Sarstedt, Etten-Leur), centrifuged (13,000 rpm, 15 min, 4 °C), and plasma was removed and stored at −40 °C until fasting metabolic hormones (leptin, insulin, resistin) were measured with a Multiplex assay according to manufacturer’s instructions (Milliplex Map Kit, mouse adipokine magnetic bead panel, Multiplex, Millipore, Amsterdam, The Netherlands). Subsequently, mice were transcardially perfused with 0.9% saline, followed by 4% paraformaldehyde in phosphate buffer (PB 0.1M, pH 7.4). Perfused white adipose tissue (WAT; gonadal (gWAT), mesenteric (mWAT), perirenal (pWAT), retroperitoneal (rWAT), inguinal (iWAT), and intrascapular brown adipose tissue (iBAT) depots were dissected and weighed. Brains were removed, postfixed, and sliced as described previously [24,38]. 

### 2.8. Immunohistochemistry

Proliferation and survival of newborn cells in the hippocampus were measured using immunohistochemistry for Ki67 and BrdU, respectively. Brain slices were mounted on precoated glass slides (Superfrost Plus slides, Menzel) and incubated with either primary antibody rat-anti-BrdU (1:500, Accurate Chemical Scientific Corporation, Westbury, NY, USA) and secondary antibody donkey-anti-rat (1:1000, Alexa 488, Invitrogen, Carlsbad, California, USA), or primary antibody polyclonal rabbit-anti-Ki67 (1:10.000, Novocastra, Amsterdam, The Netherlands) and secondary antibody biotinylated goat-anti-rabbit (1:200, Vector Laboratories, USA). The Ki67 staining procedure included an avidin-biotin complex amplification step (1:800 ABC Elite kit, Vectastain, Brunschwig Chemie, Amsterdam, The Netherlands), followed by chromogen development using 0.5mg/mL 3,3’-Diaminobenzidine. After staining, slides were coverslipped using Vectashield with DAPI (BrdU) or Antelan (Ki67). Ki67^+^ and BrdU^+^ cells in the hippocampus were quantified in coronal sections of eight matched anatomical levels along the rostrocaudal axis (bregma −1.34 until −3.80) of both hemispheres. Stereological analysis was performed as described previously [24,38]. 

### 2.9. Statistical Analysis

Data were analyzed using SPSS 20.0 (IBM software) and Graphpad Prism 8 (Graphpad software, Graphpad Holdings, LLC, La Jolla, CA, USA) and were expressed as mean ± standard error of the mean (SEM). Data were considered statistically significant when *p* < 0.05, and statistical trends were reported in case of a *p*-value between 0.05 and 0.06. Data with condition CTL/ES as predictor variable were analyzed with unpaired Student’s t-test or two-way repeated measures ANOVA. Data with condition CTL/ES and postnatal diet S-IMF/N-IMF as predictor variables were analyzed using two-way ANOVA or three-way repeated measures ANOVA. Data with condition CTL/ES, postnatal diet S-IMF/N-IMF, and adult diet SD/WSD as predictor variables were analyzed using three-way ANOVA. Post hoc analyses were performed using Tukey’s post hoc test. As multiple mice from a litter were included in experiments, litter corrections were performed when a significant contribution of litter was found in a mixed model analysis with litter included as random factor.

## 3. Results

### 3.1. ES Leads to Fragmented Maternal Care and Reduced BW Gain in Pups

**Figure 2:**
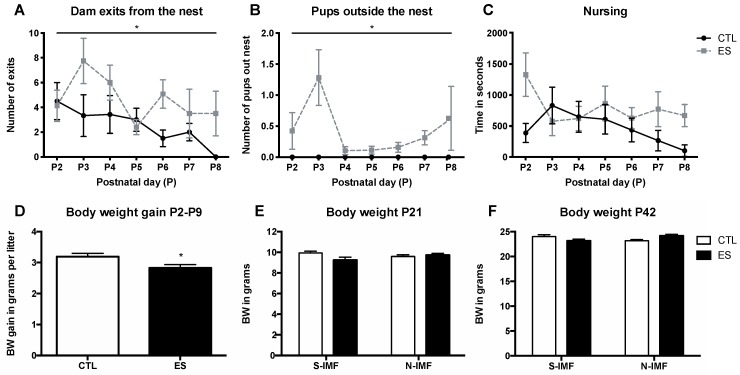
Limited nesting material results in fragmented maternal care and decreased body weight (BW) gain in pups between P2 and P9. ES dams exit the nest more often than CTL dams (**A**) and have more pups lying outside of the nest (**B**). Nursing time is equal between CTL and ES dams (**C**). BW gain in pups between P2 and P9 was decreased in ES litters (**D**). BW was normalized by P21 and not further affected by Concept Nuturis® IMF diet (N-IMF )(**E**), and no differences in BW between groups were present at P42 (**F**). Statistical analyses were performed using two-way repeated measures ANOVA for maternal care observations; independent t-test for BW gain per litter P2–P9; and two-way ANOVA for BW of pups at P21 and P42. * significant effect of condition *p* < 0.05.

The ES model led to fragmented maternal care: ES dams exited the nest more often (Figure 2A; F_condition_(1, 105) = 7.960, *p* = 0.006, F_time_(2.758, 48.27) = 1.889, *p* = 0.148, F_condition*time_(6, 105) = 1.074, *p* = 0.383) and had more pups lying outside the nest (Figure 2B; F_condition_(1, 25) = 8.021, *p* = 0.009, F_time_(0.9346, 12.46) = 2.465, *p* = 0.142, F_condition*time_(6, 80) = 2.465, *p* = 0.031) as compared with CTL dams. Total nursing time was not different between conditions (Figure 2C), indicating that ES caused fragmented but not a reduction in maternal care. Physiologically, stress in pups was reflected by a decrease in BW gain between P2 and P9 (Figure 2D; *t*(28) = 2.440, *p* = 0.021). No baseline differences in BW were present at P2 (CTL mean = 1.327 grams SEM = 0.027; ES mean = 1.391 grams SEM = 0.031; *t*(28) = 1.552, *p* = 0.132). At P21 (Figure 2E) and P42 (Figure 2F), no differences in BW were present.

### 3.2. ES and Postnatal Diet do not alter WSD-Induced Body Composition Changes 

Repeated DEXA body composition measurements could only be collected in a subgroup of mice at P42, P98, and P180 due to technical failure. As the n of this subgroup is limited, data should be interpreted with caution (see supplementary Figure S1; supplementary Figure S1D; fat percentage: F_condition_(1, 13) = 3.327, *p* = 0.093, F_postnatal-diet_(1, 13) = 3.648, *p* = 0.078, F_condition*postnatal-diet_(1, 13) = 0.201, *p* = 0.662, F_condition*time_(2, 26) = 3.186, *p* = 0.058, F_postnatal-diet*time_(2, 26) = 3.235, *p* = 0.056, F_condition*postnatal-diet*time_(2, 26) = 1.294, *p* = 0.291). 

Food intake of adult mice (P75–P165) was sampled over a period of 5 weeks. Absolute food intake was not different across experimental groups, while WSD animals had a higher caloric intake compared with SD animals (data now shown). 

### 3.3. Prolonged WSD Results in an Obesogenic Phenotype in Adulthood that is not Modulated by ES or Postnatal Diet

**Figure 3:**
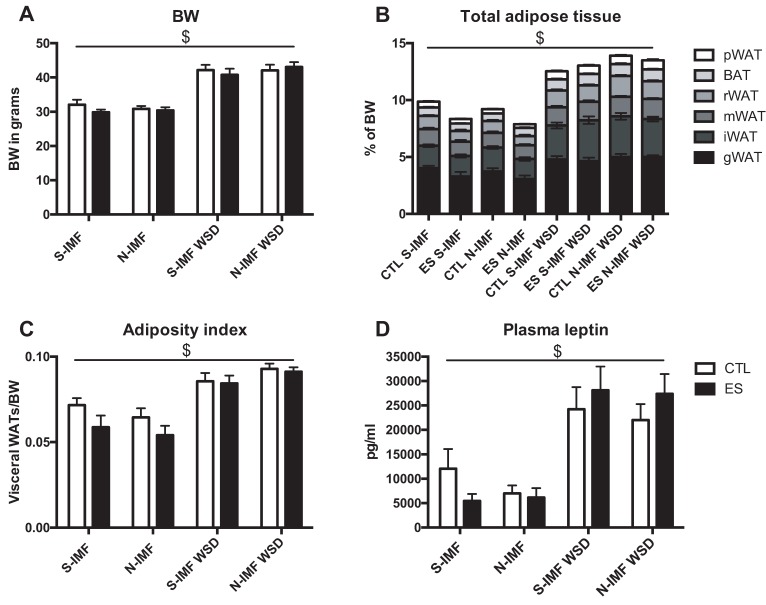
WSD increases general adiposity. In response to WSD, BW (**A**), total adipose tissue; white/brown adipose tissue (WAT/BAT) (**B**), adiposity index (**C**), and plasma leptin levels (**D**) are increased without modulation by ES or N-IMF. Statistical analyses were performed using three-way ANOVA. $ significant effect of adult diet, *p* < 0.05.

At P230, WSD resulted in an overall increase in BW, dissected adipose tissue weight, and adiposity index, and these effects were not modulated by ES or N-IMF (Figure 3A; BW: F_condition_(1, 23.508) = 1.389, *p* = 0.250, F_postnatal-diet_(1, 30.211) = 0.546, p = 0.466, F_adult-diet_(1, 38.442) = 24.146, p < 0.001, no interaction effects; Figure 3B; WAT: F_condition_(1, 24.950) = 2.708, p = 0.112, F_postnatal-diet_(1, 32.525) = 0.479, *p* = 0.494, F_adult-diet_(1, 37.528) = 7.554, *p* = 0.009, no interaction effects; Figure 3B; BAT: F_condition_(1, 24.635) = 0.682, *p* = 0.417, F_postnatal-diet_(1, 31.176) = 0.723, p = 0.402, F_adult-diet_(1, 41.271) = 6.167, *p* = 0.017, no interaction effects; Figure 3C; adiposity index: F_condition_(1, 26.951) = 3.847, *p* = 0.060, F_postnatal-diet_(1, 34.895) = 1.225, *p* = 0.276, F_adult-diet_(1, 39.752) = 4.565, *p* = 0.039, no interaction effects). The WSD-induced obesogenic phenotype was supported by elevated plasma leptin and insulin levels (Fiure 3D; leptin: F_condition_(1, 15.577) = 1.528, *p* = 0.235, F_postnatal-diet_(1, 20.183) = 0.935, *p* = 0.345, F_adult-diet_(1, 25.145) = 5.956, *p* = 0.022, no interaction effects; insulin: F_condition_(1, 18.197) = 0.155, *p* = 0.699, F_postnatal-diet_(1, 23.748) = 0.255, *p* = 0.618, F_adult-diet_(1, 26.744) = 10.957, *p* = 0.003, no interaction effects). A trend was found for WSD-induced elevated resistin levels (resistin: F_condition_(1, 13.657) = 0.034, *p* = 0.857, F_postnatal-diet_(1, 17.382) = 0.286, *p* = 0.599, F_adult-diet_(1, 24.719) = 4.030, *p* = 0.056, no interaction effects). 

### 3.4. Cognitive Function is not Altered by ES, IMF, or WSD Exposure

**Figure 4:**
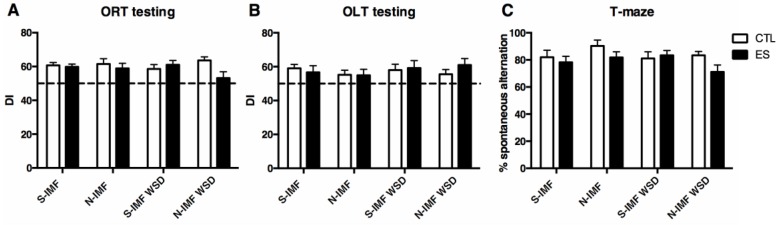
No cognitive impairment is observed in adulthood. ES, N-IMF, or WSD did not affect cognitive performance in the object recognition task (ORT) (**A**), object location task (OLT) (**B**), or T-maze (**C**). Statistical analyses were performed using three-way ANOVA.

Cognitive functioning was unaffected by ES, IMF, or WSD exposure in the current study. No differences in ORT, OLT, or T-maze performance were found between groups (Figure 4A; ORT: F_condition_(1, 80) = 2.339, *p* = 0.130, F_postnatal-diet_(1, 80) = 0.152, *p* = 0.698, F_adult-diet_(1, 80) = 0.376, *p* = 0.542, no interaction effects; Figure 4B; OLT: F_condition_(1, 76) = 0.170, *p* = 0.681, F_postnatal-diet_(1, 76) = 0.406, *p* = 0.526, F_adult-diet_(1, 76) = 0.655, *p* = 0.421, no interaction effects; Figure 4C; T-maze: F_condition_(1, 89) = 3.141, *p* = 0.080, F_postnatal-diet_(1, 89) = 0.027, *p* = 0.871, F_adult-diet_(1, 89) = 1.161, *p* = 0.284, no interaction effects). 

### 3.5. N-IMF and WSD Modulate ES-induced Effects on Neurogenesis

**Figure 5:**
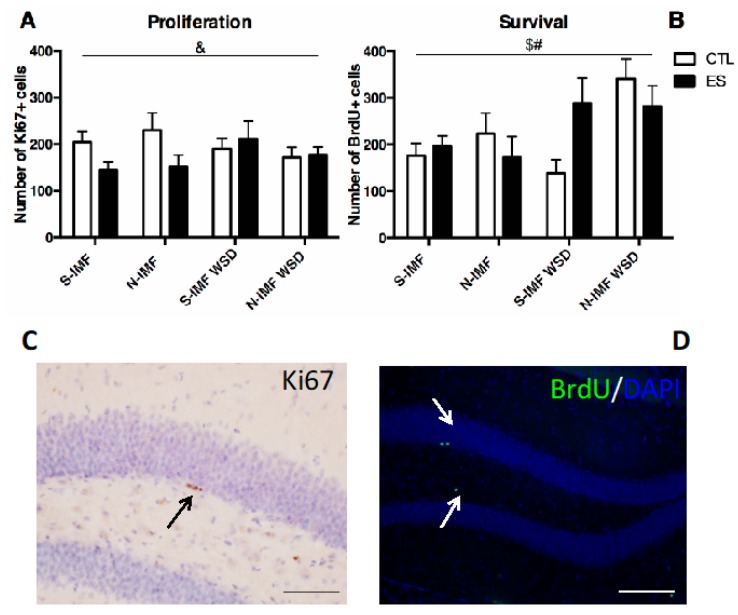
Dietary exposures modulate the effect of ES on neurogenesis. ES modulated the effect of WSD on levels of proliferating cells as measured by Ki67^+^ cells (**A**). WSD increased survival of newborn neurons and ES interacts with N-IMF in affecting the survival of newborn neurons (**B**). Statistical analyses were performed using three-way ANOVA. ^&^ interaction effect of condition and adult diet, *p* < 0.05; ^#^interaction effect of condition and postnatal diet, *p* < 0.05; ^$^ significant effect of adult diet, *p* < 0.05. Representative images of (**C**) immunohistochemistry for Ki67+ cells indicated by the black arrows and (**D**) immunohistochemistry for DAPI (blue) and BrdU (green) illustrating BrdU+ cells indicated by the white arrows in the hippocampal dentate gyrus. Scale bar: 100 μm in C and 200 μm in D.

ES and WSD interacted in affecting proliferating Ki67^+^ cells in the hippocampus, without modulation by N-IMF (Figure 5A,C; F_condition_(1, 49) = 2.461, *p* = 0.123, F_postnatal-diet_(1, 49) = 0.070, *p* = 0.793, F_adult-diet_(1, 49) = 0.062, *p* = 0.805, F_condition*adult-diet_(1, 49) = 5.551, *p* = 0.023, no other interaction effects). Further post hoc testing revealed no significant effects. 

Interestingly, ES and N-IMF interacted in affecting survival of the adult-born cells, and WSD increased survival of the adult-born cells (Figure 5B,D; F_condition_(1, 50) = 0.274, *p* = 0.603, F_postnatal-diet_(1, 50) = 3.676, *p* = 0.061, F_adult-diet_(1, 50) = 6.134, *p* = 0.017, F_condition*postnatal-diet_(1, 50) = 6.063, *p* = 0.017, no other interaction effects). Further post hoc testing revealed that N-IMF increased survival of the adult-born cells in CTL WSD mice (post hoc: CTL S-IMF WSD – CTL N-IMF WSD: *p* = 0.010), but not in ES WSD mice (no other significant post hoc effect). 

## 4. Discussion

In the current study, we showed that the protective and harmful effects of respectively N-IMF and ES on fat accumulation in response to WSD are transient and disappear after prolonged WSD exposure. Furthermore, ES and N-IMF modulated the WSD-induced response to neurogenesis, indicating that stress and diet during early life can impact adult hippocampal neurogenesis. Previous studies have demonstrated protective effects of early feeding with N-IMF on the risk of excessive body fat accumulation in response to WSD in young adult mice [35,36]. Continued WSD exposure up to P230 in the current study resulted in an increase in total WAT and BAT, a higher adiposity index, and a rise in plasma leptin and insulin levels. Interestingly, WSD-induced adiposity was not attenuated by N-IMF exposure at this age. Furthermore, while we have shown previously that ES-exposed mice accumulate more fat in response to WSD at P98 [19], this effect disappeared at P230. However, repeated DEXA body composition measurements obtained from a subgroup of animals in this study (supplementary Figure S1) revealed a pattern similar to that of earlier findings in the literature reporting an ES-related higher susceptibility to WSD-induced adiposity [16,17,18]. Moreover, this data seems to suggest an attenuation of adult WSD-induced body fat accumulation in N-IMF-exposed animals in line with previous reports [35,36,49]. Although these initial results should be interpreted with caution due to the low n, they may suggest that the increased metabolic sensitivity to WSD after ES exposure, and the protective effects of N-IMF on fat accumulation in response to WSD, are transient, and no longer present at P230. The effects of prolonged WSD might have overruled the more subtle effects of ES or N-IMF. Although the moderate WSD challenge (22% energy from fat) used in this study is mild in comparison with the often-used models of high fat diet (HFD) exposure (40%-60% energy from fat) [50,51], animals still seemed to develop a rather obesogenic phenotype, likely related to the duration of WSD exposure in the current study (~27 weeks). This is supported by a recent study showing that programming effects of N-IMF on fat deposition are transient under continuous exposure to HFD [52]. These results warrant further studies to solidify findings on programming effects of ES and N-IMF on metabolism earlier in life.

We also did not detect effects of ES, N-IMF, or WSD on cognitive functioning in the current study. This is in contrast to some of the previously reported effects of these manipulations on cognitive function [24,37,38,53]. Concerning the apparent lack of ES-induced cognitive impairments, a possible explanation could be that the postnatal diet used in the present study was provided in the form of a dough ball in the cage. This dough ball could constitute a form of environmental enrichment to the standard cage environment consisting of only sawdust bedding and paper strands. There is indeed ample evidence that environmental enrichment is able to mitigate effects of ES on cognitive function [54,55,56,57]. This is also intriguing in the light of the lack of WSD-induced cognitive impairment in our study. Yet, literature on the effects of an obesogenic diet on cognitive performance is mixed. While there are some studies showing no cognitive impairment in adult rodents exposed to HFD and tested in the ORT [58,59,60], OLT [61,62], or T-maze [63], the majority of the literature did report HFD-induced cognitive impairments for the same behavioral paradigms [64,65,66,67,68]. However, there are two other aspects of our study that differ from the studies in the literature that may explain this discrepancy. Firstly, most studies addressing the effects of an obesogenic diet on cognitive function have used a more extreme HFD (>45% fat), as compared with the modest WSD (22% fat) used in the present study. Although mice do seem to become obese on the WSD in our study, increased BW as a consequence of HFD is not always associated with impaired cognitive performance [58,59,60,62]. Secondly it should be noted that HFD might alter the status of essential fatty acids in the hippocampus, with possible consequences for cognition [69]. 

Dietary exposure to MFGM fragments derived from the membrane surrounding the lipid droplets in milk have previously been shown to positively influence cognitive function in both animals and infants [70,71]. Moreover, a previous study using the same Nuturis diet as in the present study suggested an improved adult cognitive function in healthy young adult mice as a result of early life dietary exposure to this diet [37]. In the current study, cognitive performance of CTL mice (at P120) was, however, not improved by N-IMF. This discrepancy may be explained by differences in experimental design between the studies (e.g., age of cognitive testing, housing conditions) [72]. In addition, because we did not detect cognitive impairment upon ES or WSD exposure, we consequently were unable to test our hypothesis of a possible protective effect of the Nuturis diet. 

Despite the lack of effect on cognitive functions, we observed some interesting interactions between ES and N-IMF with later-life WSD on adult hippocampal neurogenesis. The effect of ES on proliferation was modulated by adult WSD, but not by early life N-IMF exposure, while the latter happened just after ES exposure. Furthermore, in contrast to previous reports from our group where a reduction in the survival of adult-born neurons was observed [24,38], we did not find ES-induced alterations in cell survival. As suggested earlier, this effect could be due to the environmental enrichment by the dietary dough. Indeed, the unaffected survival of adult-born neurons is in line with the absence of cognitive deficits following ES in the current study, as these events were previously shown to be associated [38]. The effects of ES may also be specific to some, but not all, aspects/stages of neurogenesis (i.e., proliferation, differentiation, survival), depending on the study design [73]. Moreover, ES dysregulation of specific neurogenic parameters might be transient [74]. Our data show that ~6 month exposure to WSD does not reduce levels of neurogenesis. Although some literature reveals intact levels of neurogenesis following HFD [75,76], HFD typically disrupts neurogenesis [44,46,77,78]. Yet, such effects are mostly reported after a much shorter period of exposure (i.e., 4–8 weeks) [44,46,77,78], suggesting that decreased neurogenesis might be an initial response to HFD that normalizes over time. This is further supported by studies reporting no changes in the number of proliferating BrdU^+^ cells after 12 weeks of HFD exposure [75] or the number of differentiating DCX^+^ cells after 17 weeks [76] of HFD exposure. Also, the stem cells that give rise to neurogenesis are enriched in specific free fatty acids and share a unique fat metabolism [79]. Therefore, they may be responsive to changes in fat content of the diet, highlighting that reductions in neurogenesis following an unhealthy diet may be an initial transient response. 

When taking all experimental manipulations into consideration, a complex picture emerges. With regard to the interaction of ES and WSD on proliferation levels, it is interesting to consider whether WSD restores ES-reduced proliferation, or if the effects of ES and WSD nullify each other. A restoring effect of HFD on ES impairments has been reported more often. For instance, HFD initiated at weaning was capable of ameliorating anxiety, depressive-like behavior, and the altered stress response induced by ES [80,81,82,83]. This suggests that alterations in the hippocampus as a consequence of ES may be adaptive, allowing an organism to deal more adequately with a later adverse environment (e.g., WSD), according to the predictive adaptive response hypothesis [84,85]. Furthermore, for survival of adult-born neurons, we observed an interesting interaction between ES and N-IMF. Although no effects of ES or N-IMF were present in SD groups, N-IMF increased survival in CTL, but not ES, mice under the influence of WSD. This suggests that programming effects of N-IMF may only become apparent in response to adult WSD, or other later-life challenges. In addition, it is interesting to consider the general increase in cell survival in response to WSD. As this effect is not apparent in CTL S-IMF mice, it is likely due to an interaction of ES and/or N-IMF with WSD. Since no alterations in cognition are observed in pair with this observation, it remains inconclusive whether the WSD-induced increase in cell survival has “positive” effects, or is rather a result of, for example, dysregulated apoptosis. Furthermore, it can be considered that excess energy due to WSD has negative effects for healthy CTL mice, while it serves an adaptive purpose in ES-exposed mice (i.e., match/mismatch hypothesis). 

Further research is necessary to study the underlying mechanisms of how exactly N-IMF influences adult hippocampal neurogenesis in response to later-life WSD exposure. Possible mechanisms may include bioactive components like gangliosides and sialic acid that are present in N-IMF and known to impact neuronal growth and synaptogenesis [86]. Moreover, the altered physical structure of lipids in N-IMF affect absorption kinetics and bioavailability of lipids essential for brain development, neurogenesis, and stem cell properties [87]. Because the current results on neurogenesis were obtained more than 6 months after the N-IMF intervention ended, it is important to point out that a relatively short 26 day intervention with N-IMF during early life altered adult neurogenesis following a prolonged WSD challenge. This reinforces the powerful effects of early nutrition in determining later neurogenic capacity. 

## 5. Conclusions

We show for the first time that early life exposure to a diet containing Nuturis®, better mimicking some of the physical characteristics of lipid globules in mammalian milk, can modulate the hippocampal response to adult WSD at a structural level. Furthermore, we show that the protective and harmful effects of respectively N-IMF and ES on fat accumulation in response to WSD are transient and disappear after prolonged WSD exposure. This highlights the complexity of the interaction of the different elements investigated in this study, and stresses the importance of further investigating early dietary interventions as a potential therapeutic strategy to protect against lasting consequences of early life adversity on the brain. The current results also stress the substantial impact of a long-lasting exposure to WSD on health outcomes, a challenge also evident in current society. Adapting early life nutrition may serve as a promising noninvasive intervention to target early life adversity effects on both metabolic and mental health. However, although we provide initial evidence that early life exposure may exert programming effects on the hippocampal neurogenic response in adulthood, further studies are necessary to draw definite conclusions.

## 6. Patents

The Nuturis intervention tested is a concept that is protected by several patents, all filed before the start of the experiments reported in this study.

## Supplementary Materials

The following are available online at https://www.mdpi.com/2072-6643/12/2/570/s1, Figure S1: DEXA body composition development during WSD challenge.
