# Supplementary material for: The Effects of Early Life Stress, Postnatal Diet Modulation, and Long-Term Western-Style Diet on Later-Life Metabolic and Cognitive Outcomes
Source: Nutrients. 2020 Feb 22;12(2):570. doi: 10.3390/nu12020570 (PMC7071477; doi:10.3390/nu12020570)
Supplement: Supplementary file 1 [file nutrients-12-00570-s001.pdf]

Supplementary Materials:

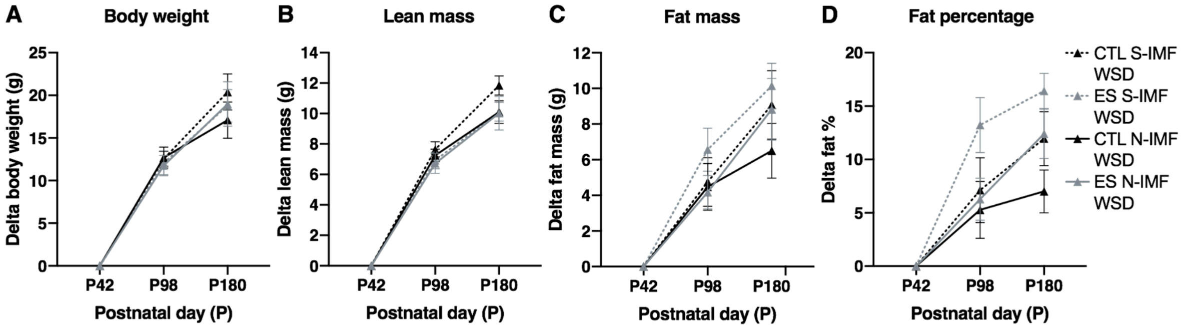

Supplementary figure S1: DEXA body composition development during WSD challenge. ES and N-IMF do not alter WSD-induced increases in delta BW (**A**), lean mass (**B**), fat mass (**C**), or fat percentage (**D**). Statistical analyses were performed using three-way repeated measures ANOVA. Absolute data points P42 represented as mean±SEM: BW: CTL S-IMF WSD 22.37±0.83; ES S-IMF WSD 22.45±0.31; CTL N-IMF WSD 23.35±0.66; ES N-IMF WSD 23.08±0.67; lean mass: CTL S-IMF WSD 17.58±0.82; ES S-IMF WSD 17.08±0.33; CTL N-IMF WSD 18.05±0.63; ES N-IMF WSD 18.20±0.54; fat mass: CTL S-IMF WSD 3.90±0.29; ES S-IMF WSD 3.32±0.22; CTL N-IMF WSD 4.90±0.22; ES N-IMF WSD 4.25±0.10; fat percentage: CTL S-IMF WSD 18.20±1.30; ES S-IMF WSD 16.38±0.43; CTL N-IMF WSD 21.38±1.06; ES N-IMF WSD 18.93±0.27.
